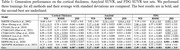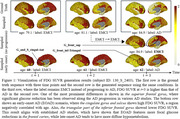# Conditional Diffusion Model using Ordinal Regression for Longitudinal Neurodegenerative Data Generation

**DOI:** 10.1002/alz70855_096933

**Published:** 2025-12-23

**Authors:** Hyuna Cho, Ziquan Wei, Seungjoo Lee, Tingting Dan, Guorong Wu, Won Hwa Kim

**Affiliations:** ^1^ POSTECH, Pohang, Korea, Republic of (South); ^2^ University of North Carolina at Chapel Hill, Chapel Hill, NC, USA

## Abstract

**Background:**

Neurodegenerative diseases like Alzheimer's disease (AD) progress irreversibly, making early detection critical. However, limited longitudinal data and irregular patient visits (e.g., 6 months to years apart) challenge modeling efforts. Recent generative methods do not consider the ordinal dynamics and temporal gaps in disease progression. Therefore, we propose a novel conditional generative model for synthesizing long‐term brain regional measurements based on disease‐relevant ordinal conditions like age and diagnostic labels.

**Method:**

We used three AD biomarkers from the Alzheimer's Disease Neuroimaging Initiative: cortical thickness (*N* = 178), Amyloid Standardized Uptake Value Ratio (SUVR) (*N* = 687), and Fluorodeoxyglucose (FDG) SUVR (*N* = 678), measured across 148 brain regions, with 2 to 10 time points per subject. Five diagnostic labels were used: Cognitively Normal (CN), Significant Memory Concern, Early Mild Cognitive Impairment, Late MCI, and AD, where the disease progresses irreversibly from CN to AD. Using age and label as independent variables, an ordinal regression model is fitted on the whole cohort to learn global patterns of disease progression. The fitted model yields multiple pseudo‐samples that fill gaps between sparse observed data points within a sequence. Afterward, a conditional diffusion model sequentially estimates the difference in consecutive pseudo‐samples to reconstruct the observed data points.

**Result:**

Table‐1 shows that our method outperformed nine generative baselines across three metrics that measure the difference between generated and test samples: Wasserstein distance (WD), Root mean squared error (RMSE), and Jensen‐Shannon divergence (JSD). In Figure 1, we qualitatively analyzed the effect of the conditions. Given a subject with three time points, the generated results using the given conditions highly resemble the ground truth. By changing the label and age, significant brain regions associated with each condition (e.g., G_front_sup and G_and_S_cingul‐Ant) exhibited variations that align with established AD studies, demonstrating the model's sensitivity to conditions.

**Conclusion:**

Our method characterizes realistic disease dynamics by incorporating ordinal regression with a diffusion model. Also, our method handles heterogeneity of sparse data with long intervals by iteratively estimating differences in pseudo‐samples yielded by the ordinal regression. Consequently, our method outperformed nine baselines, underscoring its potential to enhance our understanding of the relationships between AD progression, age, and disease stages. This work was supported by the National Research Foundation of Korea (NRF) grant funded by the Korean government (MSIT) (NRF‐2022R1A2C2092336, 90%) and RS‐2019‐II191906 (AI Graduate Program at POSTECH, 10%).